# May family routines impact oral health in American children?

**DOI:** 10.3389/froh.2024.1477036

**Published:** 2024-12-04

**Authors:** Vanessa Pardi, Giovanna Torqueto Castilho, Rachel Stewart, Huabin Luo, Wanda G. Wright, Mark Eric Moss

**Affiliations:** ^1^ School of Dental Medicine, East Carolina University, Greenville, NC, United States; ^2^ Department of Morphology and Children’s Clinic, School of Dentistry, São Paulo State University (UNESP), Araraquara, Brazil; ^3^ Department of Public Health, Brody School of Medicine, East Carolina University, Greenville, NC, United States

**Keywords:** dental caries, oral health, bedtime, sleep, mealtime, screen time, neighborhood characteristics, family

## Abstract

**Objectives:**

The present study aimed to assess the association between family routines and dental caries and self-rated oral health status.

**Methods:**

Data from the 2020–2021 National Survey of Children's Health (NSCH) completed at United States were used. Our outcome variables were self-reported dental caries (yes/no) and poor oral health condition (yes/no). Our independent variables were related to family routines: (1) Going to bed at the same time (yes/no); (2) Days having dinner together (0–7days); (3) Hours spent in front of the TV, computer, cell phone or other electronic devices (screen time); and (4) adequate sleep (yes/no). We used socioeconomic factors, health insurance coverage, family structure and neighborhood characteristics as covariates. Univariate and multiple logistic regression were used to analyze the data.

**Results:**

For the dental caries outcome, children with regular bedtimes (AOR = 0.68, 95% CI: 0.58–0.79), more frequent family dinners (AOR = 0.90, 95% CI: 0.85–0.96), and supportive neighborhoods (AOR = 0.88, 95% CI: 0.78–0.99) were less likely to report dental caries. Increased screen time (AOR = 1.10, 95% CI: 1.04–1.15) was associated with higher self-reported dental caries. For oral health status, children with regular bedtimes, (AOR = 0.60, 95% CI: 0.48–0.75), supportive neighborhoods (AOR = 0.81, 95% CI: 0.68–0.97), and with more neighborhood amenities (AOR = 0.92, 95% CI: 0.88–1.00) reported better health. More screen time (AOR = 1.11, 95% CI: 1.02–1.21) and poor neighborhood conditions (AOR = 1.13, 95% CI: 1.02–1.26) correlated with poor oral health.

**Conclusions:**

Findings from this study indicate that family routines play a significant role in children's oral health. Future research should focus on interdisciplinary family- and community-level interventions that are tailored to support healthy habits and address the needs of families.

## Introduction

Although largely preventable, dental caries is the most common childhood disease, affecting 45.8% of children aged 2–19 years in the United States (1). Globally it is estimated that 2 billion people and 514 million children have carious lesions in permanent and primary teeth, respectively (2).

Daily family routines such as mealtime spent together, sleep routines and time spent on screens can be risk or protective factors for dental caries experience. Those routines are influenced by different social determinants of health (SDoH). Studies have shown that parent's socioeconomic status (SES), health status, health behavior practices, and family composition are associated with oral health (3). The routines themselves often involve hygiene practices, such as toothbrushing, that are protective factors for oral health (4, 5). Conversely, lack of routines could be associated with oral health risk factors such as infrequent toothbrushing or increased snacking.

A wide array of family-level behaviors and practices can affect risk for dental caries, ranging from dietary factors, sleep patterns, and screentime. Families have different routine to cope with daily activities. Easy and fast meals with the consumption of ultra-processed foods, on-the-go meals, unlimited screentime to facilitate household tasks and irregular bedtime due to the number of daily tasks may influence the oral health of the family. Irregular or late bedtime or not enough hours of sleep might be considered an independent risk factor for early childhood caries (6–8). Bedtime routines that incorporate regular toothbrushing can serve as a protective factor against dental caries (9). In a study conducted with adolescents, those with an evening circadian rhythm presented with higher caries risk, seldom brushed their teeth, and ate breakfast less regularly than those with morning or normal circadian rhythm (10). Increased screen time is associated with unhealthy dietary patterns, with higher intake of cariogenic foods, such as energy-dense snacks and sugar-sweetened beverages, which contribute to dental caries (11, 12). Studies have also shown that family routines such as mealtime, media use and sleep routines influence obesity in children (13, 14). Building healthy behaviors is a daily commitment and practice. Family meal planning involves deciding what to eat and how to feed their children, while also considering the factors that influence children's eating behaviors (15).

Research has been conducted to evaluate how family routine affects several aspects of general health, however the literature of how those routines affect oral health is scarce. While prior studies have primarily focused on socioeconomic status (SES) and dental hygiene behaviors, there has been limited research on the association between family routines and dental caries. Understanding this relationship is crucial to inform future interventions aimed at improving oral health and preventing caries in children. We hypothesized that a regular bedtime routine, adequate hours of sleep, eating dinner together, and hours of screen time may influence the presence of dental caries and oral health condition in children. To test this hypothesis, we examined the relationship between family routines and both dental caries and self-rated oral health status.

## Methods

### Data source

Data were from the 2020–2021 National Survey of Children's Health (NSCH) (16). The NSCH is an annual nationally representative survey of children and youth in the age range of 0–17 years old across the 50 US states and the District of Columbia. The NSCH is funded and directed by the Health Resources and Services Administration Maternal and Child Health Bureau. The NSCH provides rich data on multiple, intersecting aspects of children's lives including sociodemographic, physical, mental, oral health, access to quality health care, and the child's family, neighborhood, school, and social context. All data were self-reported by parents/guardians. Survey respondents include parents or other caregivers familiar with the child's health and healthcare needs. The analytical sample included 86,226 children ages 2–17 years. Sampling weights were adjusted to account for nonresponse and reduce the magnitude of bias. Poststratification adjustment was conducted to ensure that sociodemographic subgroups were appropriately represented in the estimates. This study is exempt from Institutional Review Board approval as it utilizes publicly available data (17).

### Measures

#### Outcome variables

We included two self-reported outcome variables. (1) Dental caries (Yes/No) that was ascertained by answers to whether the child had decayed teeth or cavities during the past 12 months, and (2) Poor oral health (Yes/No) that was classified according to answers to the question “How would you describe the condition of this child's teeth”. We coded answers “fair/poor” as poor oral health, other answers “excellent/very good, and good” as not having poor oral health.

#### Independent variables

The family routines variables (independent variables) were (1) Going to bed at the same time (“How often does this child go to bed at about the same time on weeknights?”—2 categories, coded as 1 for answers “always/usually” and coded as 0 for answers “sometimes/rarely or never”; (2) Days having dinner together (“During the past week, on how many days did all the family members who live in the household eat a meal together?”—(0–7days); (3) Screen time “On most weekdays, about how much time does this child usually spend in front of a TV, computer, cellphone, or other electronic device watching programs, playing games, accessing the internet or using social media? (Do not include time spent doing schoolwork.”— <1 h–5 or more hours); (4) adequate sleep [“During the past week, how many hours of sleep did this child get during an average day (count both nighttime sleep and naps)”—Yes/No if whether child sleeps recommended age-appropriate hours according to American Academy of Pediatrics].

#### Covariates

Covariates were selected based on data availability and existing literature (18, 19): Age (2–5, 6–8, 9–11, 12–15, and 16–17 years), sex, racial/ethnic groups (5 groups: non-Hispanic whites, non-Hispanic blacks, Hispanics, Asians, and others), highest education of adult in the child's household (less than high school, high school, some college, and college or above), household income [in relation to Federal Poverty Level (FPL): 0%–99%, 100%–199%, 200%–399%, and 400% FPL or greater], insurance coverage (no-insurance, private, public, and both public and private), medical home (Yes/No), and children with special needs (Yes/No), family structure (living with two married parents, two not married parents, grandparents, and other caregivers), total number of children in the household, having a dental visit in the past year (Yes/No). We also explored the association between neighborhood factors and oral health. We included four neighborhood characteristics: (1) number of amenities (“In this child's neighborhood, how many amenities—parks, recreation centers, sidewalks or libraries—does it contain?” score of 0–4; (2) poor neighborhood (“In this child's neighborhood, how many detracting elements–litter or garbage on the street or sidewalk, poorly kept or rundown housing, or vandalism—are there?”—score of 0–3); (3) supportive neighborhood (“Does this child live in a supportive neighborhood?”—Yes/No); and (4) safe neighborhood (“How much do you agree that this child is safe in your neighborhood?” “yes” if answered agree or somewhat agree to the question, or “no” if answered disagree or somewhat disagree to the question).

#### Statistical analysis

First, descriptive statistics were used to characterize the study participants. Then, two multiple logistic regression models were run to assess the association between household routines and the two outcome variables—dental caries and poor oral health, controlling for other covariates. The conceptual model described by Fisher-Owen et al. (2007) was used to develop these models (18). Data analysis was conducted in Stata 16 (StataCorp). Sampling weights were incorporated into all the analyses to obtain national estimates. A significance level of *p* = 0.05 was used in this analysis.

## Results

### Descriptive statistic results

Table 1 presents the characteristics of children by the two outcome variables—self-reported dental caries and poor oral health. The prevalence of reported dental caries was 12.8% (95% CI: 12.2–13.4) and for reported poor oral health status it was 6.1% (95% CI: 5.7–6.6) in the study population (*N* = 86,226). Regarding family routines, the average number of days families ate dinner together ranged from 3.1–3.2; while screen time averaged 3.6–3.8 h for children who reported dental caries and poor oral health, respectively. The proportions of having dental caries were larger in children who did not have a regular bedtime than in children who had a regular bedtime (18.7% vs. 11.6%), who did not have adequate sleep than in children who had adequate sleep (15.1% vs. 11.6%), and had a greater mean number of days eating dinner together (3.2 vs. 3.1), had a greater mean hours of screen time in a weekday (3.6 vs. 3.4 h) in comparison to their counterparts. Covariates that had significant bivariate association with self-reported dental caries included race, family structure, number of children in the household, education level of adults in the household, family income, types of health insurance, dental visits, medical homes, and neighborhood characteristics (safety, amenities, supportive neighborhood, and poor conditions) (all *p* < .001) (Table 1).

**Table 1:** Characteristics of children by self-reported dental caries and poor oral health (*N* = 86,226).

Variables	Dental caries %			Poor oral health %		
	Yes	No	*P* value	Yes	No	*P* value
Regular bedtime			<.001			<.001
No	18.7	81.3		11.8	88.2	
Yes	11.6	88.4		4.9	95.1	
Days eating dinner together per week (Mean)	3.2	3.1	0.001	3.1	3.2	0.001
Hours of screen time (Mean)	3.6	3.4	<.001	3.8	3.4	<.001
Adequate sleep			<.001			<.001
No	15.1	84.9		8.2	91.9	
Yes	11.6	88.4		5.0	95.0	
Age (years)			<.001			<.001
2–5	9.2	90.8		4.4	95.6	
6–8	19.1	80.9		6.3	93.7	
9–11	15.6	84.4		7.2	92.8	
12–15	10.9	89.1		7.2	92.8	
16–17	10.2	89.8		5.5	94.5	
Sex			0.129			0.856
Male	13.2	86.8		6.2	93.8	
Female	12.3	87.7		6.1	93.9	
Race			<.001			<.001
non-Hispanic-white	11.3	88.7		4.5	95.5	
non-Hispanic black	12.6	87.4		7.5	92.6	
Hispanic	15.7	84.3		8.9	91.1	
Asian	13.1	86.9		6.2	93.8	
Other	12.9	87.1		5.4	94.7	
Family structure			<.001			<.001
Two parents, married	10.9	89.1		4.2	95.8	
Two parents, not married	17.9	82.1		10.0	90.0	
Single parents	15.6	84.4		8.4	91.6	
Grandparents	17.3	82.7		13.5	86.5	
Other	15.8	84.2		13.6	86.4	
SHCN^a^			<.001			<.001
No	11.7	88.3		4.9	95.1	
Yes	16.7	83.3		10.6	89.4	
Mean number of children	2.4	2.2	<.001	2.3	2.2	0.014
Highest education of adult in the child's household			<.001			<.001
<High School	18.8	81.2		12.8	87.2	
High school	15.7	84.3		9.5	90.6	
Some college	15.0	85.0		7.2	92.8	
College or above	9.6	90.4		3.1	96.9	
Income			<.001			<.001
0–99% of the Federal Poverty Level	17.8	82.2		10.5	89.5	
100–199%	15.2	84.8		8.8	91.2	
200–399%	12.5	87.5		5.7	94.3	
400%	8.4	91.6		2.1	97.9	
Insurance			<.001			<.001
No insurance	18.5	81.5		11.1	88.9	
Private	17.9	82.1		10.0	90.1	
Public only	9.2	90.8		3.3	96.7	
Both public and private	16.5	83.5		10.2	89.8	
Having a dental visit in the past year			<.001			<.001
No	10.3	89.7		9.7	90.3	
Yes	13.3	86.7		5.3	94.7	
Medical home			<.001			<.001
No	14.5	85.5		8.2	91.8	
Yes	10.8	89.2		3.7	96.3	
Neighborhood_number of_amenities (Mean)	2.5	2.6	<.001	2.5	2.6	<.001
Neighborhood in poor condition (Mean)	0.6	0.4	<.001	0.7	0.4	<.001
Supportive neighborhood			<.001			<.001
No	15.1	84.9		8.4	91.6	
Yes	10.9	89.1		4.3	95.7	
Safe neighborhood			<.001			<.001
No	17.3	82.7		12.5	87.5	
Yes	12.6	87.4		5.7	94.3	
Overall	12.8			6.1		

^a^ SHCN, special health care meeds.

There was a larger proportion of self-reported poor oral health among those who did not have a regular bedtime (11.7% vs. 4.9%), did not have adequate sleep (8.1% vs. 5.0%), had less frequent meals as a family (3.1 vs. 3.2 days/week), and had more hours of screen time (3.8 h vs. 3.4 h/weekdays) (all *p* < .001). Covariates that had significant bivariate association with self-reported poor oral health included race, family structure, number of children in the household, education level of adults in the household, family income, types of health insurance, dental visits, medical homes, and neighborhood characteristics (safety, amenities, supportive neighborhood, and poor conditions) (all *p* < .05) (Table 1).

### Logistic regression model results

Table 2 presents the results from the logistic regression models. Regarding the family routines, in the dental caries model, children who had a regular bedtime (AOR = 0.68, 95% CI: 0.58–0.79) and whose families had more dinner together (AOR = 0.90, 95% CI: 0.85–0.96) were less likely to have self-reported dental caries. On the other hand, children that had more screen time (AOR = 1.10, 95% CI: 1.04–1.15) were more likely to be reported with dental caries. Children ages 6–11 years were more likely to have dental caries than children ages 2–5 years (*p* < .001). Regarding family structure, children living in single parent families (AOR = 1.16, 95% CI 1.00–1.34) and in families with more children (AOR = 1.13, 95% CI 1.07–1.20) were more likely to report dental caries. Compared with non-Hispanic white children, Asian American children were more likely to have self-reported dental caries (AOR = 1.26, 95% CI: 1.01–1.58) while non-Hispanic black children were less likely to have dental caries (AOR = 0.76, 95% CI: 0.64–0.90). Regarding characteristics of the neighborhood, children who lived in a supportive neighborhood were less likely to report dental caries (AOR = 0.88, 95% CI 0.78–0.99) and children who lived in neighborhoods with more poor conditions were more likely to self-report dental caries (AOR = 1.13, 95% CI 1.05–1.22). Other significant factors were education level of adult in the child's household, health insurance coverage, dental visits, health special healthcare needs (SHCN), and number of children in the household (all *p* < .05).

**Table 2:** Logistic regression model results of dental caries and poor oral health.

Variables	Dental caries				Poor oral health			
	AOR	95% CI		*P* value	AOR	95% CI		*P* value
Regular bedtime	0.68	0.58	0.79	<.001	0.60	0.48	0.75	<.001
Days eating dinner together per week	0.90	0.85	0.96	<.001	0.94	0.86	1.04	0.24
Hours of screen time	1.10	1.04	1.15	<.001	1.11	1.02	1.21	0.01
Adequate sleep	0.92	0.82	1.04	0.17	0.91	0.76	1.09	0.31
Age groups (vs. 2–5 yrs)								
6–8	2.20	1.85	2.61	<.001	1.58	1.19	2.11	<.001
9–11	1.53	1.28	1.84	<.001	1.61	1.20	2.15	<.001
12–15	0.91	0.75	1.09	0.31	1.46	1.08	1.96	0.01
16–17	0.85	0.69	1.05	0.13	1.02	0.73	1.43	0.91
Female	0.91	0.82	1.02	0.11	1.05	0.89	1.24	0.56
Race (vs. NH-white)								
NH black	0.76	0.64	0.90	<.001	0.92	0.73	1.17	0.51
Hispanic	1.05	0.90	1.23	0.53	1.19	0.96	1.48	0.11
Asian	1.26	1.01	1.58	0.04	1.40	1.01	1.95	0.04
Other	1.03	0.87	1.22	0.75	0.99	0.73	1.34	0.95
Family structure (vs. two parents, married)								
Two parents, not married	1.18	0.93	1.50	0.17	1.33	0.94	1.88	0.11
Single parents	1.16	1.00	1.34	0.04	1.17	0.95	1.45	0.14
Grand parents	1.13	0.85	1.51	0.39	1.92	1.30	2.82	<.001
Other	0.95	0.61	1.48	0.83	1.36	0.80	2.32	0.26
SHCN^a^	1.32	1.18	1.49	<.001	2.10	1.78	2.48	<.001
Mean number of children in the household	1.13	1.07	1.20	<.001	1.10	1.01	1.20	0.03
Highest education of adult in the child's household (vs. < high school)								
High school	0.84	0.63	1.10	0.21	0.74	0.52	1.03	0.08
Some college	0.88	0.67	1.15	0.34	0.60	0.43	0.84	<.001
College or above	0.72	0.55	0.94	0.02	0.48	0.34	0.68	<.001
Income (vs. 0–99% Federal Poverty Level)								
100–199%	0.99	0.83	1.19	0.96	1.03	0.80	1.31	0.83
200–399%	1.00	0.82	1.21	0.98	0.97	0.74	1.28	0.84
400%	0.86	0.70	1.06	0.17	0.56	0.42	0.74	0.00
Insurance type (vs. no insurance)								
Private	0.73	0.57	0.94	0.02	0.80	0.57	1.12	0.19
Public only	0.48	0.38	0.61	<.001	0.66	0.47	0.93	0.02
Both public and private	0.67	0.50	0.92	0.01	0.93	0.61	1.43	0.76
Medical home	0.92	0.82	1.02	0.13	0.64	0.54	0.77	<.001
Having a dental visit in the past year	1.54	1.27	1.85	<.001	0.66	0.53	0.84	<.001
Neighborhood number of amenities	0.97	0.93	1.01	0.11	0.93	0.88	1.00	0.04
Neighborhood in poor condition	1.13	1.05	1.22	<.001	1.13	1.02	1.26	0.02
Supportive neighborhood	0.88	0.78	0.99	0.03	0.81	0.68	0.97	0.02
Safe neighborhood	1.19	0.90	1.57	0.22	0.94	0.66	1.33	0.72

^a^ SHCN, children and youth with special health care needs.

Similar results were found in the oral health condition model. Regarding family routines, children were more likely to present better condition if they had regular bedtime (AOR = 0.60, 95% CI 0.48–0.75) and poor condition if they had more screen time on weekdays (AOR = 1.11, 95% CI 1.02–1.21). Children ages 6–15 years were more likely to report poor oral health than children ages 2–5 years (*p* < .001). Regarding family structure, children that lived with grandparents (AOR = 1.92, 95% CI 1.30–2.82) and in families with more children (AOR = 1.10, 95%1.01–1.20) were more likely to report poor oral health. Children who lived in a supportive neighborhood (AOR = 0.81, 95% CI 0.68–0.97), with a greater number of amenities in the neighborhood (AOR = 0.92, 95% CI 0.88–1.00) were less likely to report poor oral health, while children living in a neighborhood in poor condition were more likely to report poor oral health (AOR = 1.13, 95% CI 1.02–1.26). Other significant factors were special healthcare needs (SHCN), family income level, education level of adult in the child's household, health insurance coverage, having a medical home, and had dental visits (all *p* < .05) (Table 2).

## Discussion

Family routines play a pivotal yet often overlooked role in shaping the oral health of children. Despite their potential influence, limited research has been dedicated to investigating this essential aspect of pediatric oral health. We analyzed data from the 2020–2021 National Survey of Children's Health (NSCH). The findings of this study show that household routines such as regular bedtime, having dinner together more frequently, and less screen time are associated with a decreased chance of presenting with dental caries after adjustment for other risk factors. Similar findings were found for oral health condition, where regular bedtime, less hours of screen time, families with less children living in a supportive, better condition neighborhood and with more amenities presented with better oral health condition.

Our findings are consistent with previous studies that have shown that irregular or late bedtime routines are associated with poor dental health (7–9). While a high percentage of parents report their children have a bedtime routine, there is often inconsistency with the application of routines (20). Eating and bathing are common to many routines, but other activities may vary greatly (20). Race/ethnicity, culture, and SES seem to influence the consistency of bedtime routines (20). For example, African-American children, children in Asian countries, and low SES children tend to have inconsistent bedtime routines (20). In this present study we found that regular bedtime was a protective factor for dental caries and oral health, which are consistent with prior studies.

Inconsistent bedtime routines are more apparent in families where parents work full time compared to those with stay-at-home parents or part-time employees (21). Dental caries is a multifactorial disease where oral hygiene habits that include fluoride toothpaste and diet habits play decisive roles in the development of the disease. Regular brushing and flossing to remove plaque associated with caries is one variable to maintain good oral health (22). The integration of brushing and flossing, in a consistent bedtime routine, may help to prevent caries and promote overall good oral health. Daily life tasks can be overwhelming to a family and if there is no knowledge of oral health and disease prevention, toothbrushing may not be a priority most nights. Programs designed to raise awareness about the importance of brushing before bedtime can have a positive impact on children's oral health. One such program is the American Academy of Pediatrics' (AAP) “Brush, Book, Bed” program, which links three health messages and encourages families to establish a routine where children brush their teeth, read a book, and go to bed at a regular time (23).

Children whose family had more frequent dinner together were less likely to report dental caries. However, meal frequency was not significantly associated with overall reported oral health. In a study conducted in Scottland, the authors found that family mealtime routine may influence oral health by increasing the rates of toothbrushing (5). Studies have shown that sharing family meals is associated with favorable dietary patterns (diet rich in fruits and vegetables) in children and adolescents and the frequency of those meals is correlated with less obesity, decreased risk for eating disorders and academic achievement (24). Hammons & Fiese, 2011 (25) suggested the benefits of sharing three or more family meals includes less risk of being overweight, less consumption of unhealthy foods and reduced risk of eating disorders, while at the same time increasing the odds of consumption of healthy foods. In the present data, the mean number of days eating dinner together was similar to what the authors suggested (mean = 3.2). It is important to emphasize that the AAP already recommends regular family meals to combat childhood obesity.

Prolonged use of screens has been associated with inadequate sleep, less physical activity, unhealthy eating habits, behavior problems, poor academic performances, and obesity (26–31). A recent study found that children aged 8–14 who used screens for more than 2 h daily consumed more cariogenic foods, had higher rates of tooth decay, brushed less frequently, and reported more toothaches (32). We found that children with prolonged screen use were more likely to report dental caries and poor oral health. These findings suggest that time spent using screen devices should be discussed with caregivers when educating patients on oral health.

The present study showed a higher likelihood of dental caries and poor oral health conditions when the neighborhood was classified as having more presence of detracting elements. When the caregivers classified their neighborhood as supportive, there was less likelihood that their child presented dental caries or poor oral health condition. The community also serves as an important modulator of oral health, as the social environment in which they live, such as the school the child attends and the neighborhood in which they live, can mitigate potential risks to better health (18, 33). Neighborhood context is a risk factor for dental caries (34). Parents report that facilitators within the community are based on relationships with others, useful community resources, and sharing information about oral health with others (35).

Interestingly, there seems to be an association between family structure and a child's oral health status. The risk of overall poor oral health appears to be the greatest for children under the primary care of a grandparent; as compared to children in a two-parent household, single parent household, or those with two unmarried parents. Caries rates and the self-reported poor oral health also seem to increase with more children in the home. There is approximately a 50% increase in caries and poor oral health in children living in a household with four children, as compared to children in a household with only one child. These findings should be considered when addressing oral health needs and promoting oral health practices both on the individual and community level. Further research is needed to determine how these factors influence oral health, so clinical practices and oral health education can be modified accordingly for a more profound positive effect.

There are limitations to the present study. Key data items were self-reported which brings the concern of recall bias. Second, data are cross-sectional and no causation can be assumed. Third, the data on family meals are related to the number of days that they eat dinner together, missing the knowledge on the quality of their diet. Lastly, there is no data regarding oral health hygiene routine which is a determinant factor to the development of dental caries and consequently to oral health condition. Despite these limitations, this study presents new and relevant discussion on the role of family routine, family structure and neighborhood characteristics on the oral health of children.

Dental caries is largely preventable through the use the of fluoride toothpaste, daily oral hygiene, regular dental visits, and fluoridated water. However, in cases where families lack access or do not have the knowledge on how to prevent dental caries, additional factors linked to family routine, structure, and neighborhood environments can influence oral health moving it toward health or disease. Oral health care providers have a crucial role in disseminating oral health education by empowering families with valuable and transformative knowledge. Patient-centered oral health care brings the provider and the patient/family together to work towards health (36, 37). The findings in the present study suggest that it is time to improve oral health programs and policy to focus on families and communities rather than solely on the individual risks for dental diseases.

## Conclusion

The 2020–2021 NSCH data showed that family routine and structure and the community context have a role on children's oral health. Future studies should focus on inter-disciplinary family- and community-level interventions that are tailored to support good patterns and meet the needs of families.

## Data Availability

Publicly available datasets were analyzed in this study. This data can be found here: Child and Adolescent Health Measurement Initiative (CAHMI) (2023). 2020–2021 National Survey of Children's Health (2 years combined), [(SAS/SPSS/Stata)] dataset. Data Resource Center for Child and Adolescent Health supported by Cooperative Agreement U59MC27866 from the U.S. Department of Health and Human Services, Health Resources and Services Administration (HRSA), Maternal and Child Health Bureau (MCHB). Retrieved from https://www.childhealthdata.org/help/dataset/2020-2021-combined-nsch-dataset-codebook-instruction.
